# A commentary on “DeepSeek-R1 and GPT-4 are comparable in a complex diagnostic challenge: a historical control study”

**DOI:** 10.1097/JS9.0000000000003237

**Published:** 2025-08-18

**Authors:** Li Huang, Zhaoyinqian Li

**Affiliations:** a Department of Laboratory Medicine, The Affiliated Hospital of Southwest Medical University, Luzhou, Sichuan, People’s Republic of China; b Sichuan Province Engineering Technology Research Center of Molecular Diagnosis of Clinical Diseases, Luzhou, Sichuan, People’s Republic of China; c Molecular Diagnosis of Clinical Diseases Key Laboratory of Luzhou, Luzhou, Sichuan, People’s Republic of China

*Dear Editor,*

We read with great interest the recent publication by Chan *et al*, entitled “DeepSeek-R1 and GPT-4 are comparable in a complex diagnostic challenge: a historical control study”^[1]^. In this study, the diagnostic performance of DeepSeek-R1, an open-source large language model, is compared with GPT-4 in complex cases from the New England Journal of Medicine (NEJM). DeepSeek-R1 exhibits similar performance to GPT-4 in complex medical diagnostics, with strengths in producing broader differentials. While the study highlights the potential of the open-source model for AI-assisted medical diagnosis, there are still several unresolved questions and additional perspectives that require further discussion to strengthen the reliability of its findings. This commentary is compliant with the TITAN Guidelines 2025 – governing declaration and use of AI^[2]^.

First, the author selected 100 clinicopathologic cases from NEJM, but did not describe the specific inclusion criteria for the cases. To ensure representativeness and comparability of cases, the authors should establish stringent and consistent inclusion criteria, as well as encompass a wide range of information during case selection, such as various disease types (including rare diseases), demographics (race, gender, age, and income), and diagnostic basis (imaging standards, laboratory indicators, and pathological diagnosis methods). This helps reduce systematic bias and selection bias in the model and enhances diagnostic fairness^[3]^.

Moreover, the author conducted a comparative analysis of five sets of data using DeepSeek-R1 and GPT-4, including the accuracy of final diagnoses, the inclusion rate of differential diagnoses, the length of differential diagnoses, the ranking of correct diagnoses, and the differential quality scores. However, the outcomes and underlying reasons for the comparison have not been further analyzed or explained. In particular, it remains unclear why the length of differential diagnoses generated by DeepSeek-R1 is significantly longer than that of GPT-4, yet its inclusion rate of differential diagnoses is lower. DeepSeek’s “Chain of Thought (CoT)” prompting method allows the model to progressively output its diagnostic rationale instead of giving final answers directly, thereby enhancing the traceability of its decision-making process^[4]^. This feature enables researchers to perform in-depth analysis of potential errors, pinpointing the specific stage within the reasoning chain where inaccuracies occur, and identifying both the type and underlying cause of such errors, facilitating further model optimization.

Thirdly, the study was carried out in a controlled experimental setting. The model’s performance has not been assessed in real-world conditions, potentially limiting its applicability in clinical practice^[5]^. Future studies may involve conducting prospective studies in real clinical settings, which can more accurately assess the actual utility of the model and significantly enhance its reliability in complex medical scenarios.

In conclusion, we express our gratitude to Chan *et al* for their valuable contributions to this field. The research findings indicate that DeepSeek-R1 has achieved a similar level comparable to GPT-4 in complex diagnostic tasks, and it offers the benefit of being open source and cost-effective. Its longer list of differential diagnoses may promote the expansion of clinical thinking, but it is necessary to be vigilant about the potential risks associated with the low inclusion rate. It is essential to collaborate interdisciplinary in order to optimize the model for safe, reliable, and interpretable medical AI applications in the future.

## Data Availability

None.
